# Observation of an antiferromagnetic quantum critical point in high-purity LaNiO_3_

**DOI:** 10.1038/s41467-020-15143-w

**Published:** 2020-03-16

**Authors:** Changjiang Liu, Vincent F. C. Humbert, Terence M. Bretz-Sullivan, Gensheng Wang, Deshun Hong, Friederike Wrobel, Jianjie Zhang, Jason D. Hoffman, John E. Pearson, J. Samuel Jiang, Clarence Chang, Alexey Suslov, Nadya Mason, M. R. Norman, Anand Bhattacharya

**Affiliations:** 1 Materials Science Division, Argonne National Laboratory, Lemont, IL 60439 USA; 2 Department of Physics, University of Illinois at Urbana-Champaign, Urbana, IL 61801 USA; 3 High Energy Physics Division, Argonne National Laboratory, Lemont, IL 60439 USA; 4 Department of Physics, Harvard University, Cambridge, MA 02138 USA; 5 National High Magnetic Field Laboratory, Tallahassee, FL 32310 USA

**Keywords:** Electronic properties and materials, Phase transitions and critical phenomena, Surfaces, interfaces and thin films

## Abstract

Amongst the rare-earth perovskite nickelates, LaNiO_3_ (LNO) is an exception. While the former have insulating and antiferromagnetic ground states, LNO remains metallic and non-magnetic down to the lowest temperatures. It is believed that LNO is a strange metal, on the verge of an antiferromagnetic instability. Our work suggests that LNO is a quantum critical metal, close to an antiferromagnetic quantum critical point (QCP). The QCP behavior in LNO is manifested in epitaxial thin films with unprecedented high purities. We find that the temperature and magnetic field dependences of the resistivity of LNO at low temperatures are consistent with scatterings of charge carriers from weak disorder and quantum fluctuations of an antiferromagnetic nature. Furthermore, we find that the introduction of a small concentration of magnetic impurities qualitatively changes the magnetotransport properties of LNO, resembling that found in some heavy-fermion Kondo lattice systems in the vicinity of an antiferromagnetic QCP.

## Abstract (web summary)

LaNiO_3_ is a strange metal, for reasons that are not well understood. Here, Liu et al. report evidence for scattering of charge carriers by antiferromagnetic quantum fluctuations in high-purity epitaxial thin films of LaNiO_3_, suggesting it is close to an antiferromagnetic quantum critical point.

## Introduction

In the vicinity of a quantum phase transition (QPT) near *T* = 0 K, a material can be tuned in and out of an ordered state using a parameter other than temperature, such as magnetic field or pressure^1,2^. Near a QPT, quantum fluctuations of the order parameter can have profound influence on the properties of the material, out to high temperatures^3^. For example, in a metal, quantum fluctuations can introduce long-range interactions between mobile electrons causing a breakdown of the Landau Fermi liquid (LFL)^4^, leading to a strange metal with anomalous transport and thermodynamic properties^5^. Quantum fluctuations can also mediate superconducting pairing of carriers and give rise to unusual responses to electric and magnetic fields. In rare instances, a material can be found to be intrinsically quantum critical, perched on the edge of a QPT without the need for tuning. Here we report on signatures of an antiferromagnetic (AFM) QCP in high-purity LaNiO_3_ thin films. We find that the resistivity *ρ*(*T*) shows a linear temperature dependence over almost a decade of *T* below  ~1.1 K in our cleanest samples. The linear-in-*T* resistivity crosses over to a *T*^2^ dependence in a magnetic field, consistent with the presence of AFM quantum critical fluctuations.

The rare-earth nickelates (*Re*-NiO_3_) are a widely studied family of materials that display rich electronic and magnetic properties as a result of competition between itinerancy and electron–electron and electron–lattice interactions that tend to localize carriers and give rise to an AFM insulating ground state. They have a perovskite structure, with Ni cations at the center of corner-sharing *O* octahedra. For smaller *Re* cations^6,7^, an insulating state is obtained at temperatures below *T*_MIT_ (metal–insulator transition temperature), accompanied by a structural distortion. At yet lower temperatures *T*_N_ ≤ *T*_MIT_ (*T*_N_ is the Néel temperature), an AFM state is obtained. As the *Re* cation radius increases, *T*_MIT_ decreases, merges with *T*_N_, and eventually both are driven to zero. LaNiO_3_ (LNO), with the largest *Re* cation, is the only *Re*-NiO_3_ nickelate that is metallic down to the lowest temperatures. LNO is a correlated metal^8^ and it has long been suspected that the properties of LNO are influenced by its proximity to magnetic and structural instabilities^9,10^, perhaps even by quantum fluctuations resulting from these instabilities^11,12^. However, clear experimental evidence for the effect of quantum fluctuations at low temperatures has not been reported in LNO until now.

Here, we report on systematic study on a series of LNO samples with varying degrees of disorder. The dependence of the resistivity on temperature shows agreement with theories that consider the interplay between scattering from disorder and quantum AFM fluctuations. Furthermore, we observe signatures of spin-flip scattering from localized spins in samples with greater levels of disorder at higher temperatures, while at low temperatures signatures of AFM correlations emerge, similar to behavior observed in some heavy-fermion systems. Our findings indicate that the low-temperature transport properties of LNO arise from the interplay between an AFM quantum critical point, impurity scattering and the interaction of itinerant carriers with localized spins.

## Results

### Sample preparation

Epitaxial LNO thin films are grown on (001) oriented (LaAlO_3_)_0.3_(Sr_2_AlTaO_6_)_0.7_ (LSAT) substrates by ozone-assisted molecular beam expitaxy. The growth parameters for controlling the La/Ni ratio are determined from Rutherford backscattering spectrometry (RBS) measurement (see Supplementary Fig. 1). To ensure that the oxygen vacancies are minimized, we used a high ozone flux with a background pressure of 7 × 10^−6^ torr. The growth process was monitored by reflection high-energy electron diffraction (RHEED) (see Supplementary Fig. 2). Details of the sample growth, the control of stoichiometry and X-ray characterizations are presented in “Methods” and Supplementary Fig. 3. Transport measurements were performed on six-terminal devices patterned in Hall bar geometry using photolithography. Seven samples are studied in this work, and they are labeled as LNO_#, with # being close to the residual resistivity ratio [RRR = *ρ*(300 K)/*ρ*(2 K)] of the sample. The disorder level in the sample may be characterized approximately by the residual resistivity or RRR of the sample (note that Matthiessen’s rule might not apply at *T* = 2 K due to an interplay between disorder and other scattering mechanisms).

### Resistivity measurement in the high-purity sample

**Fig. 1:**
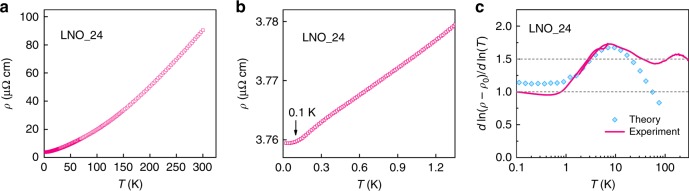
Temperature dependence of resistivity and evolution of the resistivity exponent. **a** Resistivity of LNO measured as a function of temperature from 300 K to 2 K. **b** Linear-in-temperature resistivity observed at temperatures below about 1.1 K. **c** Temperature dependence of the resistivity exponent *α* (solid line) from 300 K to 100 mK. The two horizontal dashed lines indicate exponent values of 1 and 1.5, respectively. Symbols show theoretical predictions of the resistivity exponent *α* for a clean sample (*k*_*F*_*l* ~ 1000) at AFM QCP.

Figure 1a shows the temperature dependence of the resistivity measured on sample LNO_24. This sample shows a resistivity of 3.8 μΩ cm and a mobility of about 160 cm^2^ V^−1^ s^−1^ at *T* = 2 K. The corresponding RRR is about 24, which is so far the highest RRR reported for LNO (see Supplementary Note 1 and Table 1). When the transport measurement was extended down to 25 mK, the resistivity continues to decrease without showing any flattening until about 100 mK, and in fact it shows an unexpected linear-in-temperature dependence in the temperature range 0.1 K < *T*  < 1.1 K (Fig. 1b). The same behavior in the resistivity is observed in multiple samples (see Supplementary Fig. 4). A description of the uncertainties of the data points is presented in “Methods”. Phonons are nominally not relevant in this low-temperature regime given that the Debye temperature of LNO is above 400 K^13^. Ni–O bond length fluctuations could be a source of linear—*T* resistivity at low temperatures^14^, and there may be other mechanisms as well (charge fluctuations, Umklapp scatterings). However, these mechanisms are less natural for explaining our data as they would have a weak dependence on magnetic field, which will be discussed in the following. This behavior of the resistivity in LNO is in sharp contrast with the LFL theory, which predicts a quadratic temperature dependence of resistivity at low temperatures. In Fig. 1c, we plot the resistivity exponent *α* (solid line) in *ρ*(*T*) = *ρ*_0_ + *AT*^*α*^, as a function of temperature. Here *ρ*_0_ is the residual resistivity, and *α* is calculated from $d \, {\mathrm{ln}}\,(\rho -{\rho }_{0})/d \, {\mathrm{ln}}$ (*T*). Before taking derivatives, the raw data were first smoothed using a B-Spline method. We find that *α* initially oscillates around a value of 1.5 from *T* = 300 K to about 30 K (Fig. 1c). As temperature decreases further, *α* first increases to a value of about 1.75 near *T* = 7.5 K and then begins to decrease and approaches a constant value of  ~1 in the sub-Kelvin regime.

Previous studies found that the resistivity of LNO shows a *T*^1.5^ power law at temperatures above about 30 K^8,13,15^, and anomalous exponents have been observed in other nickelates^16,17^. Similar behavior is observed here as we saw in Fig. 1c (see also Supplementary Fig. 5). A *T*^1.5^ power law scaling is often attributed to scattering by AFM spin fluctuations, as proposed by the Hertz–Millis–Moriya model for systems close to an AFM QCP^18–20^. Electronic states on the Fermi surface connected by wave vectors of the incipient AFM ordering (hot regions in *k*-space) are strongly scattered. However, these hot regions only occupy a finite phase space on the Fermi surface, and in the clean limit they are shorted out by the cold regions where such scattering does not operate^21^. It was realized subsequently that the scattering due to quantum AFM spin fluctuations can lead to a peculiar evolution^22,23^ of *α* with *T* that depends on the level of disorder. For systems close to AFM QCP in the dirty limit, a *T*^1.5^ power law should be observed over a large range of temperature. For cleaner systems (*k*_F_*l* > 10, where *k*_F_ is the Fermi wavevector and *l* is the mean free path), as temperature decreases, *α* first increases and approaches values close to 2 (symbols in Fig. 1c), as expected for LFL quasiparticles. At lower temperatures, *α* decreases continuously to a value  ~1. A signature characteristic of this model is a bump in *α* as a function of *T*^22^, which is clearly observed here at temperature *T*_Bump_ ~ 7.5 K (Fig. 1c). At even lower temperatures T < 10^−3^*T*_Bump_, which are not experimentally accessible for us, *α* increases again to the dirty limit value of 1.5 according to this theory. Using the resistivity and Hall measurements (see Supplementary Fig. 6), we estimate *k*_F_*l* ~ 500 for our cleanest samples at *T* = 2 K. Symbols in Fig. 1c are calculations reproduced from ref. ^22^ for a clean sample with *k*_F_*l* ~ 1000 (see also Supplementary Fig. 7). Further details of the analysis are presented in Supplementary Note 2.

### Restoration of LFL under a magnetic field

**Fig. 2:**
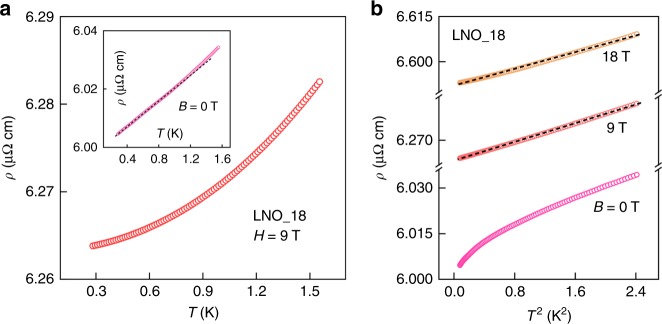
Temperature dependence of resistivity under a magnetic field. **a** Resistivity measurement under a magnetic field of 9 tesla for LNO_18. Inset shows the linear-in-temperature behavior under zero field. **b** Temperature dependence of resistivity plotted versus the square of temperature for different magnetic fields. The measurements at *B* = 9 and 18 tesla show a linear dependence on *T*^2^.  Note the positive magnetoresistance. The standard deviation of the mean at each point is smaller than the symbol size. The black dashed lines in **b** and the inset of **a** are straight guidelines.

If AFM fluctuations near a QCP are responsible for the linear *ρ*(*T*) that we observe at low temperatures, an applied magnetic field may be used to suppress these fluctuations and restore the LFL. Such behavior has been observed in heavy-fermion compounds close to a QCP—for example in YbRh_2_(Si_1−*x*_Ge_*x*_)_2_ and CeCoIn_5_^24,25^. Figure 2a shows our measurement results on LNO_18 under a magnetic field of 9 T, presenting a quadratic dependence of resistivity on temperature, i.e., *ρ*(*T*) = *ρ*_0_ + *AT*^2^. Inset of Fig. 2a shows the linear *ρ*(*T*) in zero magnetic field in the sub-Kelvin regime, as has been observed in other clean samples. Thus, a restoration of LFL behavior in LNO is observed under a magnetic field. This is seen more clearly in Fig. 2b, where we plot *ρ*(*T*) vs *T*^2^ at different fields. The data can be fit well to a *T*^2^ dependence for *B* = 9 and 18 T, with corresponding *A* coefficients in *ρ*(*T*) being 8 × 10^−3^ and 7 × 10^−3^ μΩ cm K^−2^, respectively. These values of *A* are about 3–4 times larger than those, ~2 × 10^−3^ μΩ cm K^−2^, reported for LNO previously^13,15,26^, which is suggestive of enhanced scattering near the QCP. We note that this crossover to LFL in the resistivity under a magnetic field is seen only in samples with RRR ≥ 18. Therefore, the presence of disorder in LNO could detune the system away from the QCP.

### Interplay of AFM spin fluctuations and disorder

**Fig. 3:**
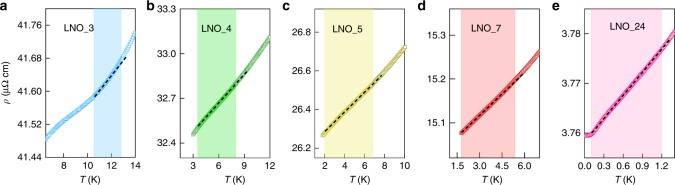
Resistivity of samples with different disorder levels. **a**–**e** Low-temperature measurement of resistivity on samples with different impurity levels. The shaded region in the plot indicates the temperature range where the resistivity shows a linear temperature dependence (quasilinear for low-RRR samples). The black dashed lines are straight guidelines. A downward curvature in the resistivity is observed in these samples near the low-temperature end *T* ≤ *T*_low_.

To examine the role of disorder on the quantum critical behavior of LNO, we grew a series of samples under different ozone pressure, substrate temperatures, and with slight variance in La/Ni ratio (<2%). The growth conditions of each sample are presented in Supplementary Table 2. The main form of impurities introduced during the growth are oxygen vacancies and Ni^2+^, which also act as local magnetic moments. The sample with the highest RRR was grown under the highest effective ozone pressure, a substrate temperature of 615 ^∘^C, and a La/Ni ratio very close to 1. Figure 3 shows the resistivity measurement on these samples. As sample become cleaner (increasing RRR from left to right), there is a corresponding change in the temperature range (shaded region, from *T*_low_ to *T*_high_) over which a linear *ρ*(*T*) appears. In cleaner sample, *T*_low_ decreases, while the temperature ratio *T*_high_∕*T*_low_ over which we observe linear *ρ*(*T*) increases. This behavior is consistent with the Rosch’s model^23^ where *T*_low_ ~ 1∕*k*_*F*_*l* and ${T}_{{\rm{high}}}/{T}_{{\rm{low}}} \sim \sqrt{{k}_{F}l}$ (see Supplementary Note 2).

We found that at lower temperatures (*T* ≤ *T*_low_), the resistivity shows anomalous sublinear temperature dependence. This is identified as a downward curvature in the resistivity curve, which is clearly seen in Fig. 3a at *T* < 10 K. Similar behaviors in the resistivity have been observed in heavy-fermion Kondo lattice systems. In CeCo_*x*_Rh_1−*x*_In_5_ compounds, the appearance of a sublinear *ρ*(*T*) was associated with the formation of short-range AFM order together with Kondo coherence, which are intimately related to an AFM QCP. Short-range AFM order may also emerge in LNO at low temperatures. This is corroborated by measurements in a magnetic field, which suppresses weak AFM order. With an applied magnetic field of 9 T, the sublinear part of *ρ*(*T*) at low temperatures is mostly quenched (see Supplementary Fig. 8a). As the sample’s RRR becomes higher, both the magnitude and the onset temperature of the sublinear part of *ρ*(*T*) becomes smaller. In our cleanest sample (shown in Fig. 3e or Fig. 1b), a small sublinear *ρ*(*T*) can be identified only at very low temperatures (<300 mK), suggesting that pure LNO is in close proximity to an AFM QCP. We note that a small sublinear *ρ*(*T*) has also been observed at low temperatures in high-quality bulk single-crystal LNO (Supplementary Fig. 8b).

### Scattering from local magnetic moments

According to current understanding, nickelates are negative charge-transfer materials^7,27,28^ where the nominally 3*d*^7^-states of the Ni^3+^ sites take one electron from oxygen leading to a $3{d}^{8}\underline{L}$ electron configuration, where $\underline{L}$ stands for a ligand hole on the *O* 2*p* orbitals. This may be seen (approximately, due to Ni–O covalence) as a localized 3*d*^8^ magnetic moment (*S* = 1) at a Ni impurity site^29^ that is partially screened by the ligand hole which takes on the *e*_*g*_ symmetry of the 3*d*^8^ orbitals. The Fermi level lies in the strongly hybridized *O* 2*p* band, with the lowest lying excitations being from 2*p* (filled) to 2*p* (empty) states in the continuum. Such low lying excitations have been inferred^30^ from resonant X-ray inelastic scattering measurements in NdNiO_3_, and the dominance of holes in magnetotransport and thermogalvanic properties has also been experimentally demonstrated^31^ recently for LNO. The screening and hybridization between the conduction charge carriers and localized magnetic moments in LNO bear resemblance to an underscreened Kondo lattice^32^. Thus, when an electron is doped into LNO, it would fill a ligand hole $\underline{L}$ and create an extra unscreened 3*d*^8^ moment localized on the Ni site, which can act like a magnetic scattering center.

**Fig. 4:**
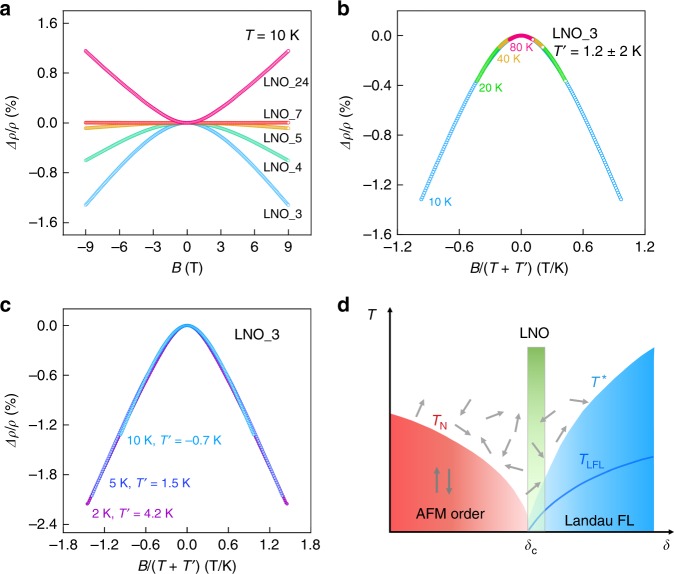
Magnetotransport measurements and phase diagram. **a** Longitudinal magnetoresistance measurements for a series of samples at 10 K. **b**, **c** The negative LMR measured on LNO_3 at temperatures above and below 10 K, respectively. The *x*-axis is scaled by $B/(T+T^{\prime} )$ as discussed in the text. **d** Phase diagram of a system with Kondo lattice character. Green rectangle represents the position where LNO is likely to sit. T^*^ marks the temperature at which Kondo coherence starts to develop. *T*_LFL_ represents the temperature below which LFL behavior appears, such as a quadratic temperature dependence of the resistivity. Small arrows with random orientations represent local magnetic moments.

In our samples, we observe a systematic evolution of the magnetoresistance with impurity levels. Figure 4a shows our longitudinal magnetoresistance (LMR) measurement results. The magnetic field is in the film plane and parallel to the current direction, which minimizes orbital MR effects from the Lorentz force. The LMR changes gradually from negative to positive as the RRR increases from 3.3 (LNO_3) to 24 (LNO_24). In samples, such as LNO_3, showing a large negative LMR, the magnitude of the LMR does not depend on the direction of the magnetic field (see Supplementary Fig. 9). The resistivity also shows metallic behavior down to the lowest temperature. These phenomena could not be explained by a weak localization mechanism^33^. Furthermore, for *T* ≥ 10 K, the normalized MR defined as $\Delta \rho /\rho =[\left(\rho (B,T)\right.-\rho (0,T)]/\rho (0,T)$, can be made to collapse by plotting *Δρ*∕*ρ* vs. $B/(T+T^{\prime} )$, allowing for a variance (±2 K) in the free parameter $T^{\prime}$, as shown in Fig. 4b (note that ${T}^{\prime}\ll T$ in this temperature range). In particular, $\Delta \rho /\rho \sim -{[B/(T+T^{\prime} )]}^{2}$, in agreement with predictions for negative MR induced by spin-flip scattering from localized impurities^34^. Here, the overall $B/(T+T^{\prime} )$ scaling can be understood as the temperature dependence of the magnetic susceptibility *χ* of single ions; $T^{\prime}$ is a measure of correlation among them, analogous to the Curie–Weiss temperature. At lower temperatures (*T* < 7 K), shown in Fig. 4c, while *Δρ*∕*ρ* ~ −*B*^2^ still holds, the negative LMR begins to saturate, and $T^{\prime}$ increases as *T* decreases approximately as $T^{\prime} \sim$ (6.2 K − *T*). Essentially, the denominator in $B/(T+T^{\prime} )$ remains constant at low temperatures. This behavior is similar to the Curie–Weiss law for an antiferromagnet, where the magnetic susceptibility remains finite when the AFM correlation increases at low temperatures (see Supplementary Figs. 10 and 11). Crucially, the temperature at which the negative LMR starts to saturate matches the temperature where the *ρ*(*T*) becomes sublinear. These magnetotransport measurements, therefore, further support that pure LNO is in the vicinity of an AFM QCP; while the introduction of magnetic impurities results in short-range AFM ordering (see Supplementary Note 3). It has been shown that only a small amount of oxygen vacancies in LNO, which promotes the formation of Ni^2+^ sites, can result in long-range magnetic ordering^35^.

## Discussion

The transport property of LNO shows similarities to those reported for Kondo lattice or heavy-fermion materials^36^. For instance, CeCoIn_5_ shows a linear *ρ*(*T*) at low temperatures, which becomes quadratic in a magnetic field. The magnetic field dependence of resistivity also shows a $\Delta \rho /\rho \sim -{[B/(T+T^{\prime} )]}^{2}$ dependence. Thus, the low-temperature physics of LNO might involve a subtle interplay of AFM quantum fluctuations and Kondo physics^37–40^. Figure 4d shows a proposed phase diagram containing LNO. As the tuning parameter *δ* increases, the system undergoes a transition from an AFM phase (under *T*_N_) to an LFL state. Here, *δ* can be the *Re* cation radius or strain. The green rectangle represents where LNO is likely situated in this phase diagram, near the critical point and on its right side. It is understood from this phase diagram that LNO can be driven from strange metal towards an LFL by an external magnetic field upon suppressing AFM fluctuations near the QCP. At elevated temperatures, local magnetic moments give rise to single-impurity spin-flip scattering, particularly for samples with a higher degree of disorder. Recently, superconductivity has been observed in an infinite-layer nickelate^41^, which might have a connection to the QCP that we observe here in LNO. This possibility warrants further exploration.

In summary, we have observed non-LFL behavior at low temperatures in high-purity epitaxial thin films of LNO. In particular, a linear-in-temperature dependence of resistivity is observed that extends over almost a decade of temperature at *T* ≤ 1.1 K in our cleanest samples. The evolution of the resistivity exponent as a function of temperature follows model predictions for a system with three dimensional quantum critical AFM fluctuations and weak impurity scattering. These fluctuations are suppressed in a magnetic field, and the LFL is restored. These results, and the systematics of single-impurity scattering in samples with elevated disorder level suggest that high-purity LNO is on the verge of an AFM quantum phase transition.

## Methods

### Sample growth details

When the effusion cells containing La and Ni sources are heated to deposition temperatures ( ~1430 °C for La and  ~1290 °C for Ni), atoms are evaporated toward the substrate at a stable rate. The deposition rate is measured by a quartz crystal microbalance (QCM) before and after growth. To calibrate the QCM measurement, we deposit La and Ni on a MgO substrate using the same growth condition as for the actual samples. We then use RBS to determine the relative ratio of La to Ni. Using a MgO substrate makes the background near La and Ni peaks clean in the RBS measurement. Shown in Supplementary Fig. 1 is the analysis of the RBS data, which give a ratio of La to Ni of about 1.009. This value was then used to adjust the shutter times of La and Ni sources during growth to target a nominal La/Ni ratio to as close to one as possible. In our lowest resistivity samples, the drift in La and Ni rates during growth were under 0.3% h^−1^.

Supplementary Table 2 presents detailed growth parameters of the samples discussed in the main text. The growth temperature was measured from a thermocouple. Elemental sources of La and Ni were evaporated sequentially from effusion cells using a block-by-block technique. The layer sequence was LaO–NiO_2_–LaO.... The growth process was monitored by RHEED. Shown in Supplementary Fig. 2a is the RHEED intensity measured as a function of time as growth proceeds. When the LaO layer is deposited the surface becomes rough, which results in a drop in RHEED intensity as shown in Supplementary Fig. 2b. The deposition of a NiO_2_ layer makes the sample surface smooth enhancing the RHEED intensity, which is shown in Supplementary Fig. 2c. We note that RHEED intensity oscillations can have complex origins^42^. Therefore, one period of a RHEED oscillation corresponds to one unit-cell of LaNiO_3_. In Supplementary Fig.  2c, half ordering peaks can be clearly identified, which indicates good surface crystallinity of the LaNiO_3_ film. We observed that although the high-RRR samples have good surface crystallinity, the surface roughness is higher than those low-RRR samples grown at lower temperatures. Therefore, the sample’s resistivity does not positively correlate with the sample’s roughness. Supplementary Fig. 2d shows the characterization of the surface roughness on sample LNO_18 using X-ray reflectivity (XRR) measurement. The roughness of this high-RRR sample is about 0.86 nm, which is higher than that of LNO_3 of about 0.38 nm determined from the same measurement. After growth, the film’s thickness and *c*-axis parameter were characterized by low-angle XRR and X-ray diffraction (XRD) measurements, respectively (see Supplementary Fig. 3). The film thickness obtained from fitting the XRR data was  ~30.8 nm, which is in good agreement (<1% error) for a 80-unit cell LNO with the *c*-axis lattice constant of  ~3.82 Å  obtained from the XRD.

### Device fabrication and measurement details

Standard photolithography was used to fabricate Hall bar devices used in the transport measurement. To prevent the formation of oxygen vacancies, liquid-nitrogen cooling was used during Ar-ion milling. Electrical contacts were made by depositing 50-nm thick platinum using sputtering. The channel of the Hall bar has a dimension of 50 × 800 μm^2^, and the distance between the two voltage contacts is 400 μm. Low-noise transport measurements were performed using a Lakeshore model 372 AC resistance bridge, Stanford lock-in amplifiers and Keithley 6221/2182A current source/nanovoltmeters in delta mode. Typical excitation currents of  ~2 μA were used in our measurements. Cooling of electrons to temperatures below 100 mK was achieved by multistage filtering of the measurement lines in a dilution fridge.

### Uncertainties of the measurement

In all figures including those in the Supplementary Information, the data points for the resistivity measurement are interpolations or mean values of the raw data which have higher density than those shown in each plot. The error bar, representing the standard deviation of the mean at each data point, is smaller than the symbol size. In these measurements, the uncertainty in resistivity is ~8 × 10^−4^ μΩ cm. There is a systematic uncertainty (<10%) in resistivity that comes from variations in the dimensions of individual device, which does not affect the RRR value of the sample.

## Supplementary information

Supplementary Information

Peer Review File

## Data Availability

All data presented in the main text and all resistivity data in the Supplementary Information are available for download at the following url: 10.5061/dryad.zpc866t5v.

## References

[1] Coleman P, Schofield AJ (2005). Quantum criticality. Nature.

[2] Gegenwart P, Si Q, Steglich F (2008). Quantum criticality in heavy-fermion metals. Nat. Phys..

[3] Sachdev, S. *Quantum Phase Transitions* (Cambridge University Press, 2009).

[4] Schofield AJ (1999). Non-fermi liquids. Contemp. Phys..

[5] Stewart GR (2001). Non-fermi-liquid behavior in *d*- and *f*-electron metals. Rev. Mod. Phys..

[6] Torrance JB, Lacorre P, Nazzal AI, Ansaldo EJ, Niedermayer C (1992). Systematic study of insulator-metal transitions in perovskites *R*NiO_3_ (*R* = Pr, Nd, Sm, Eu) due to closing of charge-transfer gap. Phys. Rev. B.

[7] Catalano S (2018). Rare-earth nickelates *R*NiO_3_ : thin films and heterostructures. Rep. Prog. Phys..

[8] Zhou J-S, Marshall LG, Goodenough JB (2014). Mass enhancement versus stoner enhancement in strongly correlated metallic perovskites: LaNiO_3_ and LaCuO_3_. Phys. Rev. B.

[9] Hoffman JD (2016). Oscillatory noncollinear magnetism induced by interfacial charge transfer in superlattices composed of metallic oxides. Phys. Rev. X.

[10] Fabbris G (2018). Emergent *c* -axis magnetic helix in manganite-nickelate superlattices. Phys. Rev. B.

[11] Allen SJ (2015). Gaps and pseudogaps in perovskite rare earth nickelates. APL Mater..

[12] Subedi A (2018). Breathing distortions in the metallic, antiferromagnetic phase of LaNiO_3_. SciPost Phys..

[13] Zhang J, Zheng H, Ren Y, Mitchell JF (2017). High-pressure floating-zone growth of perovskite nickelate LaNiO_3_ single crystals. Cryst. Growth Des..

[14] Rivadulla F, Zhou J-S, Goodenough JB (2003). Electron scattering near an itinerant to localized electronic transition. Phys. Rev. B.

[15] Guo H (2018). Antiferromagnetic correlations in the metallic strongly correlated transition metal oxide LaNiO_3_. Nat. Commun..

[16] Liu J (2013). Heterointerface engineered electronic and magnetic phases of NdNiO_3_ thin films. Nat. Commun..

[17] Mikheev E (2015). Tuning bad metal and non-fermi liquid behavior in a mott material: rare-earth nickelate thin films. Sci. Adv..

[18] Moriya, T. *Spin fluctuations in itinerant electron magnetism*, Vol. 56 (Springer Science and Business Media, 2012).

[19] Millis AJ (1993). Effect of a nonzero temperature on quantum critical points in itinerant fermion systems. Phys. Rev. B.

[20] Löhneysen Hv, Rosch A, Vojta M, Wölfle P (2007). Fermi-liquid instabilities at magnetic quantum phase transitions. Rev. Mod. Phys..

[21] Hlubina R, Rice TM (1995). Resistivity as a function of temperature for models with hot spots on the fermi surface. Phys. Rev. B.

[22] Rosch A (1999). Interplay of disorder and spin fluctuations in the resistivity near a quantum critical point. Phys. Rev. Lett..

[23] Rosch A (2000). Magnetotransport in nearly antiferromagnetic metals. Phys. Rev. B.

[24] Custers J (2003). The break-up of heavy electrons at a quantum critical point. Nature.

[25] Paglione J (2003). Field-induced quantum critical point in CeCoIn_5_. Phys. Rev. Lett..

[26] Son J (2010). Low-dimensional mott material: transport in ultrathin epitaxial LaNiO_3_ films. Appl. Phys. Lett..

[27] Johnston S, Mukherjee A, Elfimov I, Berciu M, Sawatzky GA (2014). Charge disproportionation without charge transfer in the rare-earth-element nickelates as a possible mechanism for the metal-insulator transition. Phys. Rev. Lett..

[28] Green RJ, Haverkort MW, Sawatzky GA (2016). Bond disproportionation and dynamical charge fluctuations in the perovskite rare-earth nickelates. Phys. Rev. B.

[29] Park H, Millis AJ, Marianetti CA (2012). Site-selective mott transition in rare-earth-element nickelates. Phys. Rev. Lett..

[30] Bisogni V (2016). Ground-state oxygen holes and the metal-insulator transition in the negative charge-transfer rare-earth nickelates. Nat. Commun..

[31] Liu C (2019). Counter-thermal flow of holes in high-mobility LaNiO_3_ thin films. Phys. Rev. B.

[32] Lee S, Chen R, Balents L (2011). Metal-insulator transition in a two-band model for the perovskite nickelates. Phys. Rev. B.

[33] Scherwitzl R (2011). Metal-insulator transition in ultrathin LaNiO_3_ films. Phys. Rev. Lett..

[34] Peski-Tinbergen TV, Dekker A (1963). Spin-dependent scattering and resistivity of magnetic metals and alloys. Physica.

[35] Wang B-X (2018). Antiferromagnetic defect structure in LaNiO_3−*δ*_ single crystals. Phys. Rev. Materials.

[36] Malinowski A (2005). c-axis magnetotransport in CeCoIn_5_. Phys. Rev. B.

[37] Doniach S (1977). The kondo lattice and weak antiferromagnetism. Physica B+C.

[38] Coqblin B, Nunez Regueiro MD, Theumann A, Iglesias JR, Magalhães SG (2006). Theory of the kondo lattice: competition between kondo effect and magnetic order. Philos. Mag..

[39] Si Q, Steglich F (2010). Heavy fermions and quantum phase transitions. Science.

[40] Karner VL (2019). Local metallic and structural properties of the strongly correlated metal LaNiO_3_ using ^8^Li *β*–NMR. Phys. Rev. B.

[41] Li D (2019). Superconductivity in an infinite-layer nickelate. Nature.

[42] Sun HY (2018). Chemically specific termination control of oxide interfaces via layer-by-layer mean inner potential engineering. Nat. Commun..

