# Supplementary material for: Observation of an antiferromagnetic quantum critical point in high-purity LaNiO3
Source: Nat Commun. 2020 Mar 16;11:1402. doi: 10.1038/s41467-020-15143-w (PMC7075863; doi:10.1038/s41467-020-15143-w)
Supplement: Supplementary file 1 — Supplementary Information [file 41467_2020_15143_MOESM1_ESM.pdf]

**Supplementary Information:**

# "Observation of an antiferromagnetic quantum critical point in high-purity $LaNiO_3$"

Changjiang Liu,[1] Vincent F. C. Humbert,[2] Terence Bretz-Sullivan,[1] Gensheng Wang,[3] Deshun Hong,[1] Friederike Wrobel,[1] Jianjie Zhang,[3] Jason Hoffman,[4] John E. Pearson,[1] J Samuel Jiang,[1] Clarence Chang,[3] Alexey Suslov,[5] Nadya Mason,[2] M. R. Norman,[1] and Anand Bhattacharya[1]

[1]Materials Science Division, Argonne National Laboratory, Lemont, IL 60439
[2]Department of Physics, University of Illinois at Urbana-Champaign, Urbana, IL 61801, USA
[3]High Energy Physics Division, Argonne National Laboratory, Lemont, IL 60439, USA
[4]Department of Physics, Harvard University, Cambridge, MA 02138, USA
[5]National High Magnetic Field Laboratory, Tallahassee, FL 32310, USA

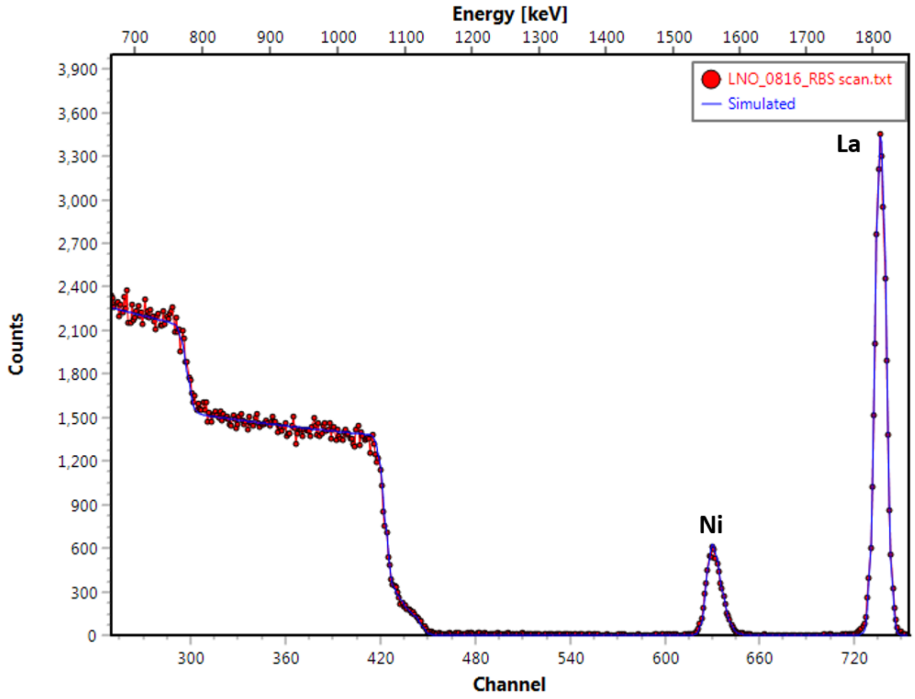


**Supplementary Figure 1. Rutherford backscattering spectrometry (RBS) measurement.** The blue line is a simulation of the data (red) which is used to find the relative concentration of La and Ni for the control sample.

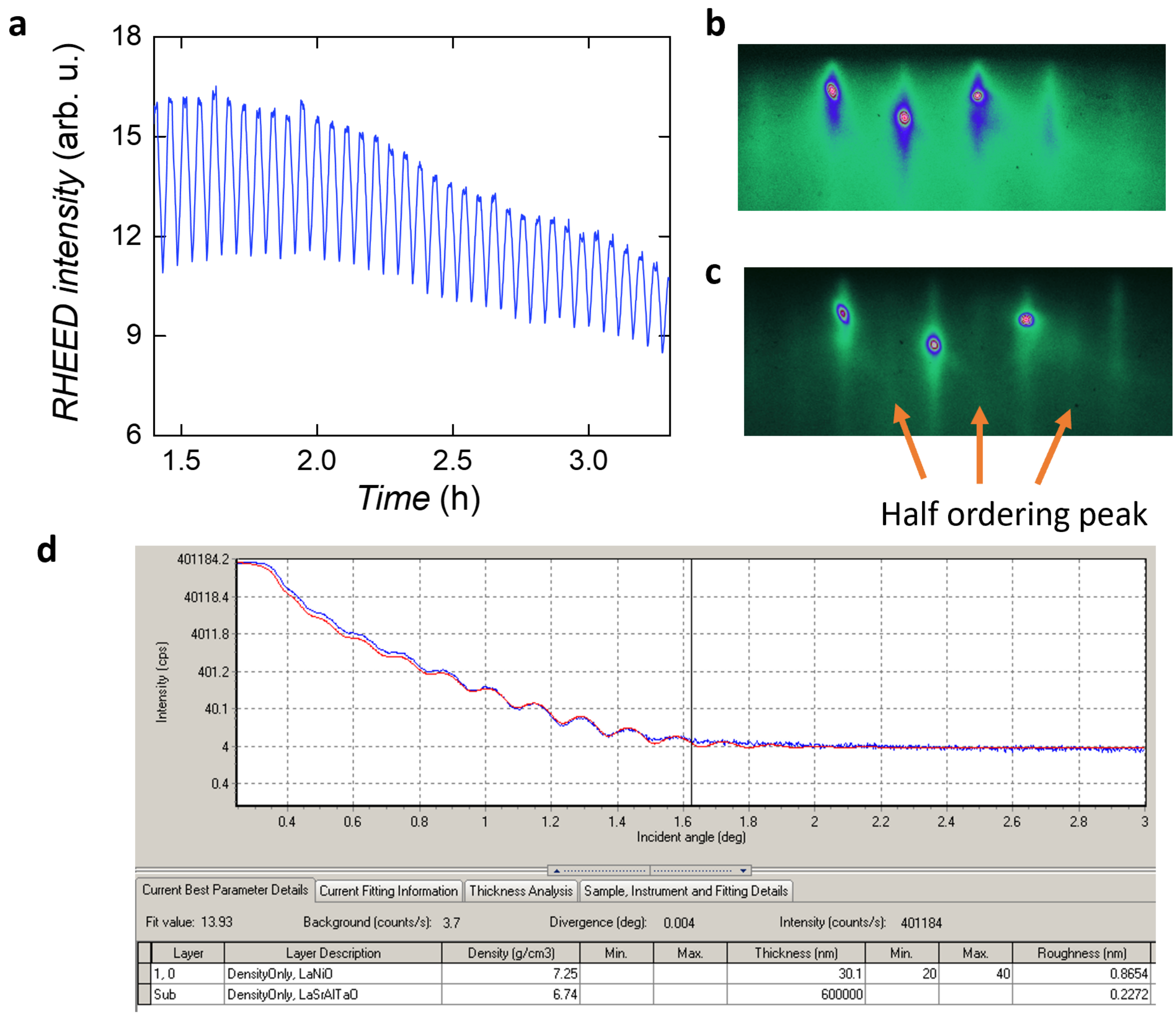


**Supplementary Figure 2. Reflection high-energy electron diffraction (RHEED) pattern and surface characterization. a** RHEED intensity as a function of time during growth. **b** and **c**, RHEED image on the screen after the deposition of a LaO and $NiO_2$ layer, respectively. **d** Low-angle X-ray reflectivity measurement show that the surface roughness of the high-RRR sample (LNO_18) is about 0.86 nm.

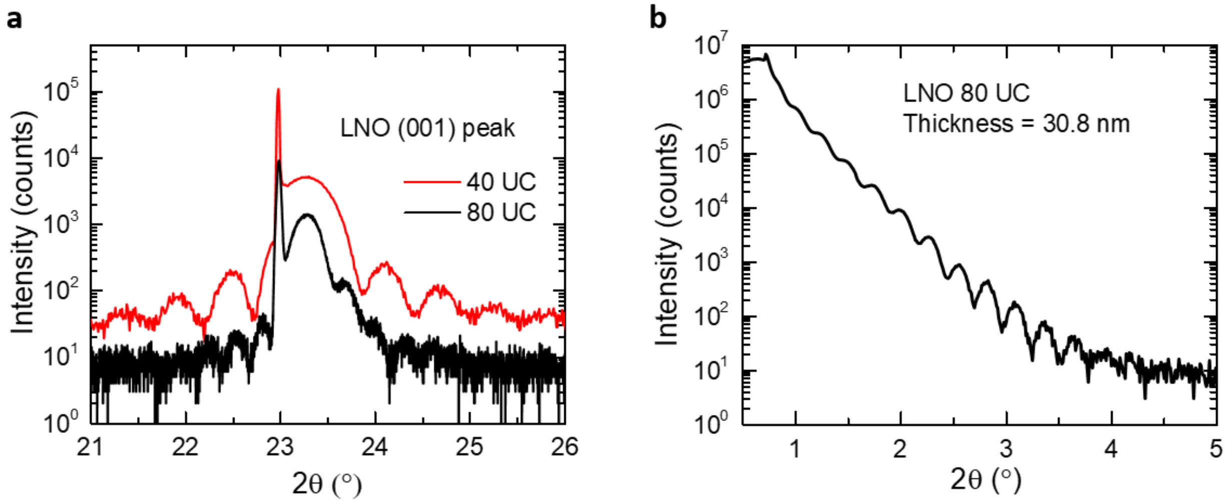


**Supplementary Figure 3. X-ray characterizations. a** X-ray diffraction measurements for 40- and 80-unit cell (UC) samples. **b** Low angle X-ray reflectivity measurement for determining the thickness of the film. Figure is reproduced with permission from ref. 31 in the main text, with permission from the American Physical Society.

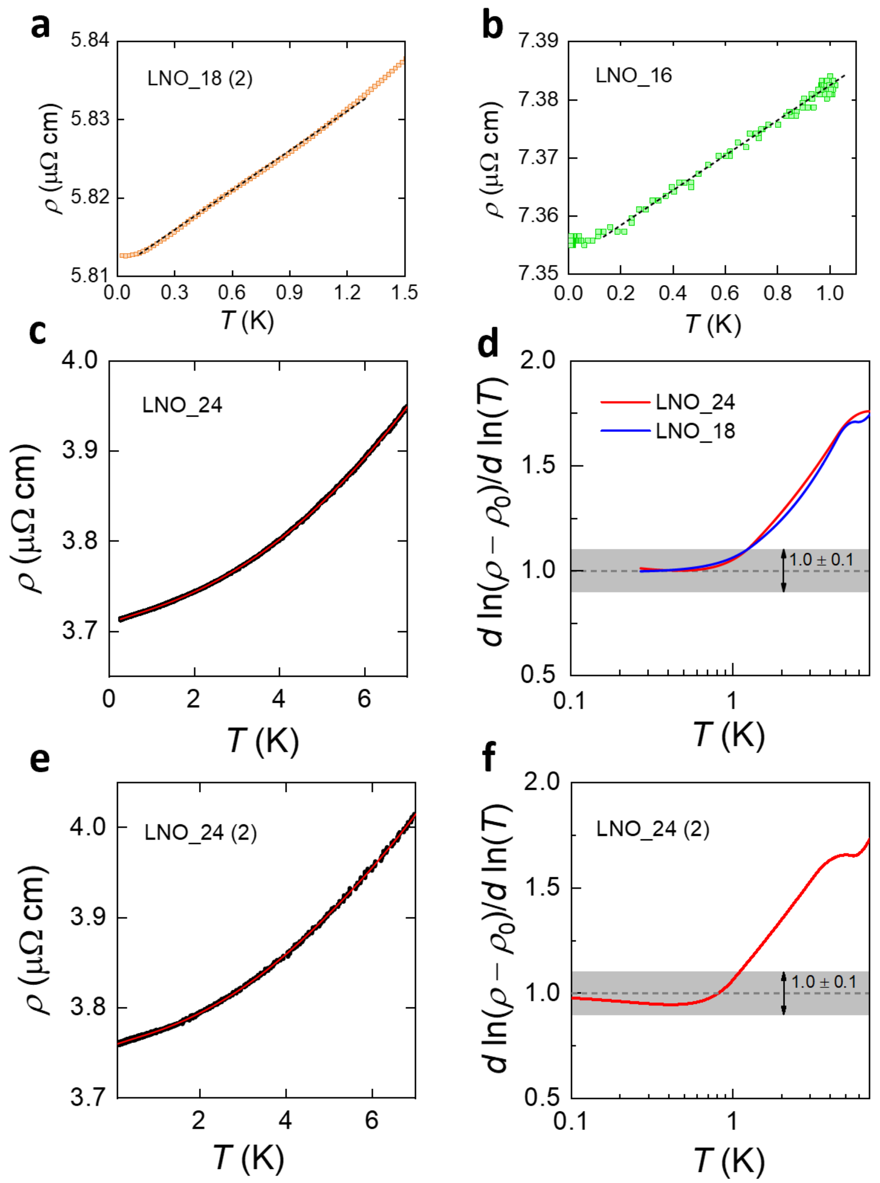


**Supplementary Figure 4. Resistivity measurements of different samples using different cryostats. a** LNO_18 (2) measured in Nadya Mason's group at UIUC. **b** LNO_16 measured in a dilution cryostat in the material science division at Argonne National Laboratory, which shows similar linear-in-temperature of resistivity as other samples. Data points in (**b**) are raw data, which has less density than those in other measurements. **c** Resistivity measurement in sample LNO_24 using a helium-3 cryostat at the MagLab, Tallahassee. **d** Analysis of resistivity exponent for LNO_24 and LNO_18. The shaded region indicates an exponent value of $1 \pm 0.1$. **e** Resistivity measurement for sample LNO_24 (2) using dilution refrigerator at UIUC. **f** Analysis of resistivity exponent for LNO_24 (2), which is also presented in Fig. 1 of the main text. Red solid line in (**c**) and (**d**) are interpolation of the data, which is used to determine the resistivity exponent.

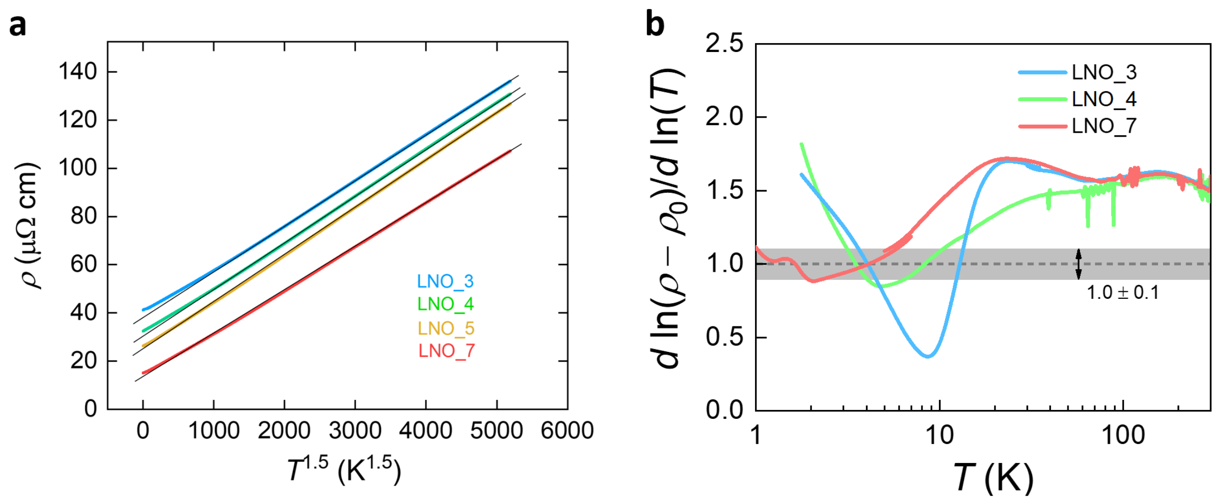


**Supplementary Figure 5. Transport measurement on LNO samples with higher residual resistivities. a** All of the samples show a resistivity exponent of about 1.5 in the higher temperature range, similar to that in the high-RRR sample discussed in the main text. **b** Analysis of the resistivity exponent shows that these samples display sublinear behavior (exponent less than 1) and a quasilinear behavior (shaded area) in a relatively narrow temperature range.

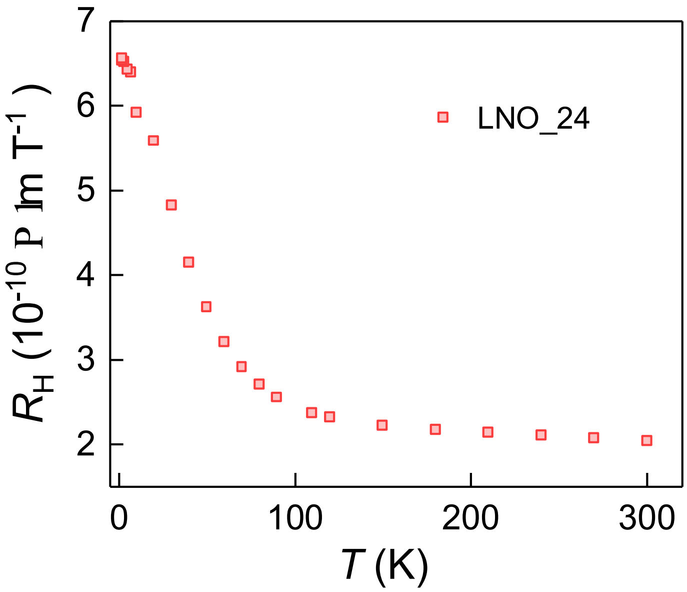


**Supplementary Figure 6. Hall coefficient measured at different temperatures for LNO_24.** The Hall coefficient shows an enhancement at temperatures below about 100 K. The uncertainty in each data point is smaller than the symbol size.

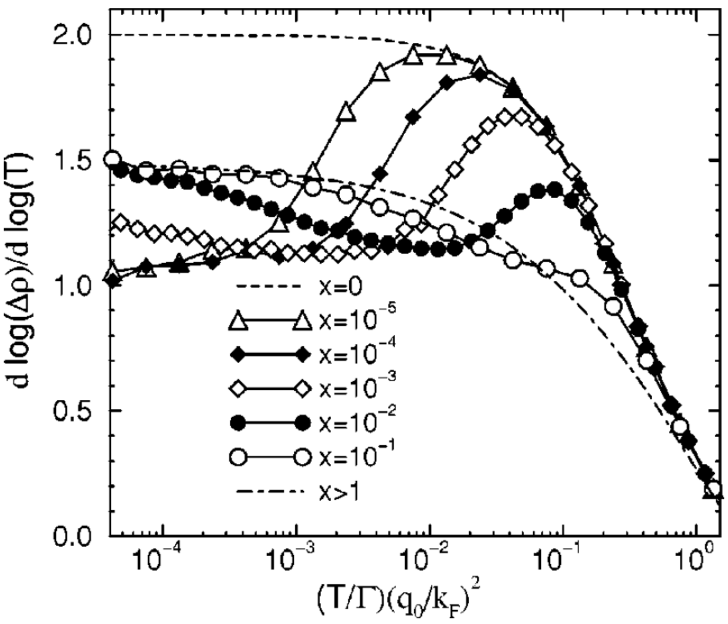


**Supplementary Figure 7. Theoretical prediction of the resistivity exponent at the AFM QCP.** $q_0$ and $\Gamma$ (~ $T_{SF}$) are characteristic momentum and energy scales for the spin-fluctuations. Figure reproduced from ref. 15 in Supplementary Information, with permission from the American Physical Society.

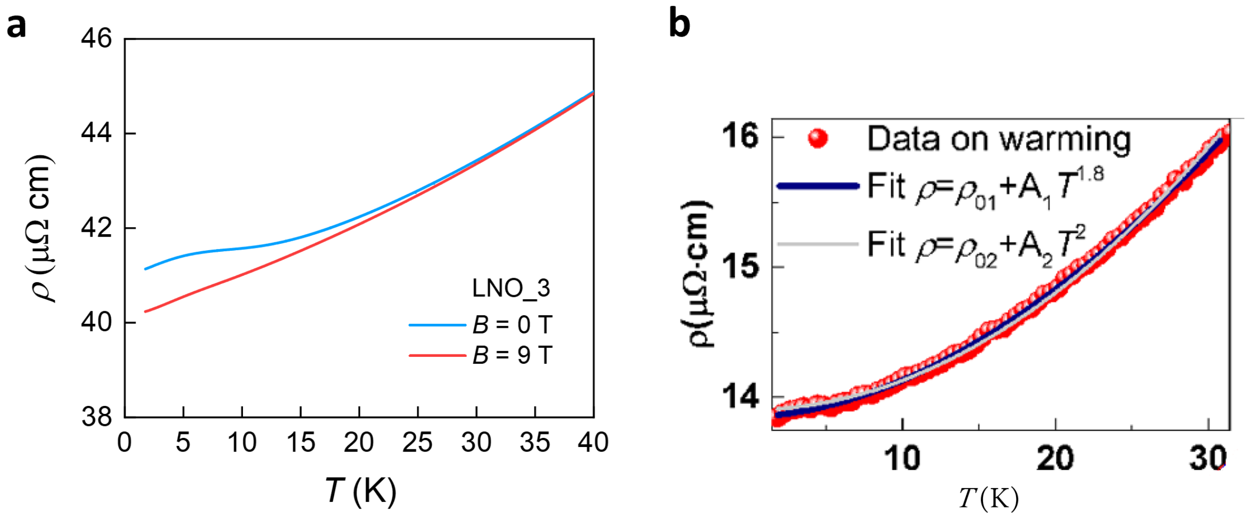


**Supplementary Figure 8. Suppression of the sublinear $\rho$(T) and resistivity of a bulk LNO sample. a** The large sublinear $\rho$(T) measured in LNO_3 at low temperatures is largely suppressed by a magnetic field of 9 tesla. **b** Small sublinear $\rho$(T) can be identified in bulk single crystal LNO at T < 5 K. Here, the sample is grown using a high-pressure floating zone method[10]. The plot is adapted from ref. 10 in Supplementary Information with permission from the American Chemical Society.

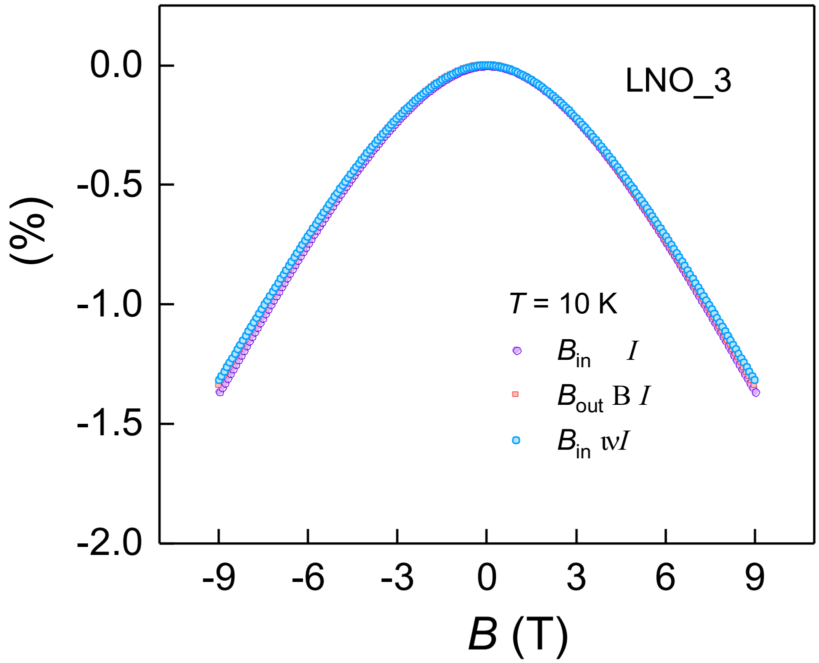


**Supplementary Figure 9. Negative MR measurement with different field orientations.** $B_{in}$ and $B_{out}$ refer to in-plane and out-of-plane field. In all three field orientations, the size of the negative MR is the same.

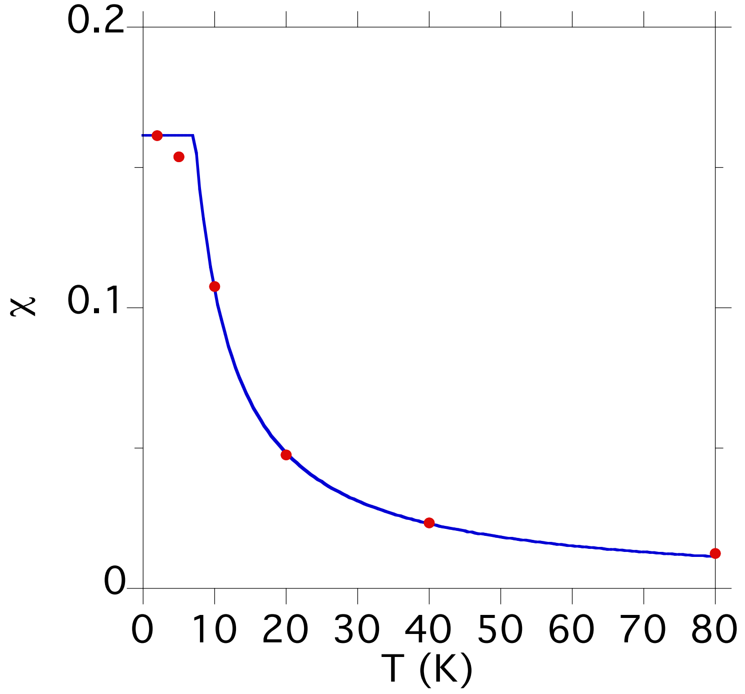


**Supplementary Figure 10. Plot of $\chi$ versus $T$**. Here, $\chi$ is extracted from $\Delta\rho/\rho = -\chi^2 H^2$. The red dots are the data and the blue curve is a fit of the form $1/(T-T_{CW})$ to the last four data points, resulting in a $T_{CW}$ of 1.8 K. This form is then assumed to saturate below 7 K to the value represented by the first data point.

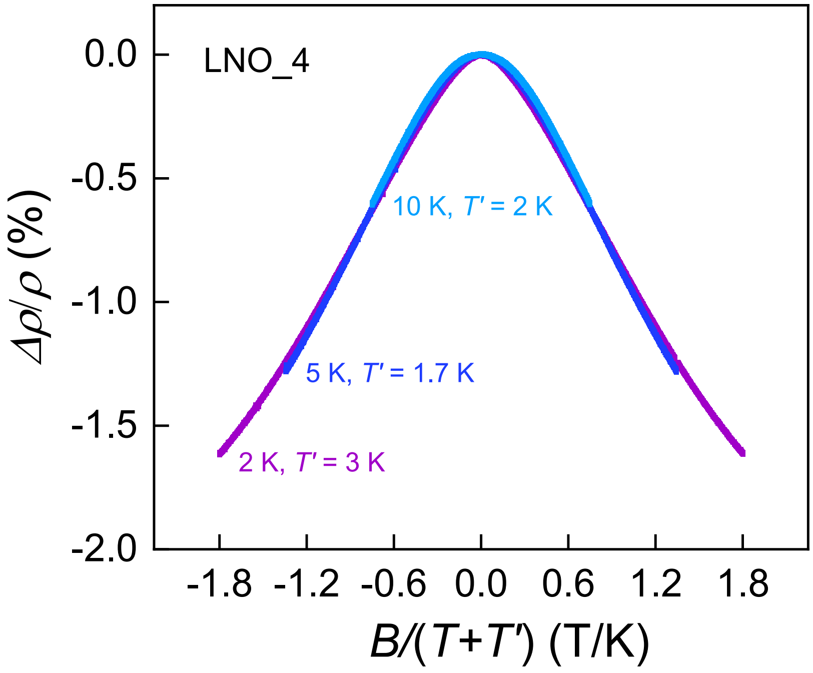


**Supplementary Figure 11. Scaling plot of the negative MR for sample LNO_4 at T ≤ 10 K.** The magnitude of the negative MR for this sample starts to saturate at a lower temperature (< 5 K) than that of LNO_3 (~ 7 K). Correspondingly, the sublinear part of $\rho(T)$ for this sample sets in at lower temperature of around 3.5 K.

| Group | *Substrate* | *Thickness* | *Growth technique* | $\rho_{300K}$ (μΩ cm) | RRR |
|---|---|---|---|---|---|
| Gayathri *et al.* | | bulk | | 790 | 6 |
| Xu *et al.* | | bulk | | 380 | 11 |
| Zhou *et al*. | | bulk | | 105 | 16 |
| Zhang *et al.* | | bulk | | 125 | 9 |
| Guo *et al.* | | bulk | | 90 | 15 |
| Scherwitzl *et al.* | STO | ~ 30 unit cell | Sputtering | 270 | 2.7 |
| Son *et al.* | LSAT<br>$LaAlO_3$ | ~ 80 unit cell<br>~ 25 unit cell | Sputtering | 150 | ~ 2.5 |
| King *et al.* | $LaAlO_3$ | 25 unit cell | MBE | 140 | ~ 11 |
| Wrobel *et al.* | LSAT | 25 unit cell | MBE | 90 | 18 |
| **This work** | **LSAT** | **80 unit cell** | **MBE** | **90** | **24** |

**Supplementary Table 1. Comparison of LNO samples from different groups.**

| Sample # | Growth T (℃) | Ozone nozzle distance | Nominal La/Ni ratio | La Drift (%/h) | Ni Drift (%/h) | Actual La/Ni ratio | No. unit cells | Termination layer | RRR |
|---|---|---|---|---|---|---|---|---|---|
| **LNO_3** | 575 | 13 cm | 1.01 | - 1.04 | 0.8 | 0.991 | 40 | LaO | 3.3 |
| **LNO_4** | 575 | 13 cm | 0.998 | - 1.28 | 0.14 | 0.984 | 40 | LaO | 4 |
| **LNO_5** | 575 | 13 cm | 1.01 | 0.11 | 0.1 | 1.010 | 40 | $NiO_2$ | 4.8 |
| **LNO_7** | 575 | 13 cm | 0.997 | - 0.14 | - 0.04 | 0.995 | 80 | $NiO_2$ | 7.3 |
| **LNO_18** | 615 | 9 cm | 1 | 0.26 | - 0.19 | 1.010 | 80 | $NiO_2$ | 18 |
| **LNO_24** | 615 | 8 cm | 1 | -0.18 | - 0.15 | 0.999 | 80 | $NiO_2$ | 24 |

**Supplementary Table 2. Growth condition of the samples**. Nominal La/Ni ratio was the targeted ratio. Actual ratio accounts for drift in deposition rate.

## Supplementary Note 1: Background of $LaNiO_3$ samples

In earlier work, $LaNiO_3$ films grown on $SrTiO_3$ substrates using ozone-assisted molecular beam epitaxy (MBE) and a layer-by-layer growth technique showed low temperature resistivities $\rho$(2 K) ~ 30 μΩ cm,[1] and a residual resistivity ratio (RRR) approaching ~ 3, consistent with values measured in bulk ceramic samples[2]. Epitaxial LNO films have also been grown using reactive sputtering[3,4] with comparable or higher values of $\rho$(2 K) and lower RRR, while PLD grown films have higher resistivities[5,6,7]. In more recent years, several groups have reported on the MBE growth of fully oxygenated LNO with lower resistivities[8], where in particular the Stuttgart group showed that for LNO/LSAT $\rho$(2 K) < 5 μΩ cm with RRR values ~ 18 could be obtained using layer-by–layer ozone–assisted MBE.[9] Concurrently, high-quality bulk single crystals of $LaNiO_3$ have been grown using high pressure synthesis techniques[10], where RRR values ~ 15 and $\rho$(2 K) ~ 6 μΩ cm have been reported[11], significantly lower than previous values. These developments point to the need for mitigating oxygen vacancies that can act as point defects and donors of electrons which may drive Ni to a lower oxidation state. Supplementary Table 1 summarizes the resistivities of LNO grown by different groups.

## Supplementary Note 2: Analysis – interplay of AFM fluctuations and disorder

In the vicinity of a quantum critical point (QCP) for an antiferromagnet in three dimensions, quantum spin fluctuations can give rise to non-Fermi liquid behavior[12]. In particular, the AFM QCP spin density wave type fluctuations give rise to hot lines for scattering on some parts of the Fermi surface, and in the dirty limit, $\rho(T) \sim T^{3/2}$. However, in the clean limit[13] these regions are shorted out by the normal regions of the Fermi surface, giving rise to $\rho(T) \sim T^2$. In samples with intermediate disorder, $\rho(T) \sim T^{\alpha}$ ($1.5 < \alpha < 2.0$) at high $T$, and $\sim T^{3/2}$ at very low $T$.[14,15] Furthermore, a linear $\rho(T) \sim \sqrt{x}T$ may be observed in the crossover regime at intermediate temperatures, over a range $\frac{1}{k_{\mathrm{F}}l} < \frac{T}{T_{\mathrm{SF}}} < \frac{1}{\sqrt{k_{\mathrm{F}}l}}$ , where $k_{\mathrm{F}}$ is the Fermi wavevector, $l$ is the mean free path, $x$ is the impurity level which is proportional to $\frac{1}{k_{\mathrm{F}}l}$, and $T_{\mathrm{SF}}$ is a characteristic energy scale for the AFM spin fluctuations. $k_{\mathrm{F}}l$ can be estimated by $k_{\mathrm{F}}l = k_{\mathrm{F}}v_{\mathrm{F}}\tau = k_{\mathrm{F}}\frac{\hbar k_{\mathrm{F}}}{m^*}\frac{m^*}{e^2 n\rho_{xx}}$, in which $v_{\mathrm{F}}$ is Fermi velocity, $\tau$ is the scattering time in Drude model, $\hbar$ is the reduced Planck constant, $e$ is the elementary charge and $n$ is the charge carrier density. Using the measured Hall coefficient (Supplementary Figure 6) and resistivity at $T$ = 2 K, $k_{\mathrm{F}}l$ is estimated to be about 500 for our cleanest sample. We have assumed a single band to calculate the carrier concentration $n$, and a spherical Fermi surface for calculating $k_{\mathrm{F}}$.

The linear-in-temperature regime of resistivity in LNO_24 extends over almost a decade of temperature, consistent with this estimate. The corresponding $T_{\mathrm{SF}}$ is about 12 K. As $k_{\mathrm{F}}l$ decreases with increasing disorder (Fig. 3 of the main text), the magnitude of $T$ at which the crossover regime with linear $\rho(T)$ emerges increases, while the ratio of $T_{\mathrm{high}}/T_{\mathrm{low}}$ shrinks to well under a decade in more disordered samples, as expected from this model.

Theoretical data points in Figure 1c of the main text were reproduced from the results shown in Supplementary Figure 7 for $x = 10^{-3}$. In LNO, spin-density-wave fluctuations with a wavevector $q_0 = (1/4,1/4,1/4)$ are most likely present[16], noting that $q_0$ is the ordering wavevector seen in other $Re$NiO$_3$ materials. However, the effective value of $\Gamma/q_0^2$ obtained from the fit of the prediction to the experimental results shown in Fig. 1c of the main text is a factor of 10 larger than $T_{\mathrm{SF}}/q_0^2$. The $k_{\mathrm{F}}$ is taken as $\pi/a$, where $a$ is the length of unit cell. We note that a similar discrepancy exists in analyzing the results of $CeNi_2Ge_2$ (ref. 15), which is also a quantum critical metal.

The extracted $T_{SF}$ of about 12 K is too low for the $\rho(T) \sim T^{1.5}$ behavior that we observe in the range 30 K < $T$ < 300 K in our cleanest samples. In fact, the $T^{1.5}$ scaling is also seen in samples with less purity, as shown in Supplementary Figure 5. This suggests that the $T^{1.5}$ behavior in this higher temperature range may not originate from magnetic fluctuations. Recently, both theory[17,18] and experiment have found a strong presence of bond length fluctuations in LNO[19]. These bond fluctuations have been suggested to result in a $T^{1.5}$ dependence of the resistivity[20]. Further theoretical work is needed to address this robust $T^{1.5}$ phenomenon in LNO, that has been reported by several groups.

## Supplementary Note 3: Negative MR – Single-impurity Kondo scattering, quantum critical magnets

The magnetotransport measurement shows a predominant negative MR on less clean LNO samples. To investigate the mechanism, we took the measurement with different field orientations. Shown in Supplementary Figure 9 is the negative MR measured on LNO_3. The magnitude of the negative MR shows little dependence on the field orientation. For a weak localization effect, the out-of-plane field would produce a larger negative MR than the in-plane field, particularly when the localization length (which is significantly larger than the mean free path) is larger than the film thickness. Under these circumstances (which do hold for our cleaner films) the out-of-plane magnetic field is substantially more efficient in breaking the phase coherence of the localized electronic states than an in-plane field. Thus, this would give rise to a highly anisotropic negative MR. In contrast, the negative MR we observe is isotropic.

In the main text, the negative MR is explained through the single-impurity Kondo scattering. The scattering centers comes from isolated magnetic impurities, such as $Ni^{2+}$ with $S = 1$. In addition to the $B/(T+T')$ scaling plot presented in the main text, single-impurity scattering also produces a quadratic magnetic field dependence in the low field regime. This is indeed observed in our measurements, whereas weak-localization effects do not have this type of field/temperature dependence.

In a simplified model considering spin-flip scattering arising from localized magnetic moments[21], the following expression holds for the resulting negative MR:

$$\frac{\rho(H)}{\rho(0)} \approx 1 - \left(\frac{g\,\mu_B\,H}{k_B T}\right)^2 \left[\frac{J^2}{V^2+J^2S(S+1)}\frac{1}{6}S(S+1) + \left\{\frac{2VJ}{V^2+J^2S(S+1)}\frac{1}{3}S(S+1)\right\}^2\right]. \quad (1)$$

Here, $H$ is the magnetic field, $g$ is the Landé g-factor, $k_B$ is Boltzmann constant, $J$ is the exchange coupling strength between the conduction electrons and the localized magnetic moments, $V$ is the elastic scattering potential from impurities and $S$ is the magnitude of the local moment. $H/T$ scaling with a quadratic dependence is a central feature of this model.

The observed behavior can also be related to quantum criticality. For the single-ion Kondo case, the magnetoresistance $\Delta\rho$ goes as $-M^2_{imp}$ (coming from the low field expansion of a cosine

function, $M_{imp}$ is the impurity magnetic moment)[22]. As $M_{imp}$ goes as $\chi H$, where $\chi$ is the susceptibility, then $\Delta\rho \sim -\chi^2 H^2$. This result is general for quantum critical magnets[23] where it is often expressed as $\Delta\rho \sim -\xi^4 H^2$ where $\xi$ is the correlation length with $\chi \sim \xi^2$. At the mean field level, $c \sim 1/(T\text{-}T_{CW})$ where $T_{CW}$ is the Curie-Weiss temperature, consistent with the functional form we used to fit the MR with $T_{CW} \sim -T'$ (Supplementary Figure 10). Note that $T_{CW}$ can be non-zero even if $T_N$ is zero, as is the case at the quantum critical point. We find that the MR data deviates from this form at low temperatures. We observe a trend towards saturation of the MR as $T$ falls below some characteristic low $T$ scale. This saturation can be due to a variety of causes: deviation from the quantum critical point in the less clean samples, the correlation length exceeding the film thickness, or other effects associated with the deviation of the low-$T$ resistivity from quantum critical behavior.

## Supplementary References


[1] A. Yu Dobin, K. R. Nikolaev, I. N. Krivorotov, R. M. Wentzcovitch, E. D. Dalhlberg and A. M. Goldman, "Electronic and crystal structure of fully strained $LaNiO_3$ films", *Phys. Rev. B* **68**, 113408 (2003).

[2] J.-S. Zhou, J.B. Goodenough, B. Dabrowski, P.W. Klamut, and Z. Bukowski, "Probing the metal insulator transition in Ni(III)-oxide perovskites", *Phys. Rev. Lett.* **84**, 526 (2000).

[3] J. Son et al., "Low-dimensional Mott material: Transport in ultrathin epitaxial $LaNiO_3$ films", *Appl. Phys. Lett.* **96**, 062114 (2010).

[4] R. Scherwitzl, P. Zubko, C. Lichtensteiger, J.-M. Triscone, "Electric-field tuning of the metal-insulator transition in ultrathin films of $LaNiO_3$", *Appl. Phys. Lett.* **95**, 222114 (2009).

[5] K. Horiba, R. Eguchi, M. Taguchi, A. Chainani, A. Kikkawa, Y. Senba, H. Ohashi, and S. Shin, "Electronic structure of $LaNiO_{3-x}$: An *in situ* soft x-ray photoemission and absorption study", *Phys. Rev. B* **76**, 155104 (2007).

[6] M. Zhu *et al.*, "Effect of composition and strain on the electrical properties of $LaNiO_3$ thin films", *Appl. Phys. Lett.* **103**, 141902 (2013).

[7] E. J. Moon *et al.*, "Strain-dependent transport properties of the ultra-thin correlated metal $LaNiO_3$", *New J. Phys.* **13**, 073037 (2011).

[8] P. D. C. King *et al.*, "Atomic-scale control of competing phase in ultrathin $LaNiO_3$", *Nat. Nanotechnol.* **9**, 443 (2014).

[9] F. Wrobel *et al.*, "Comparative study of $LaNiO_3/LaAlO_3$ heterostructures grown by pulsed laser deposition and oxide molecular beam epitaxy", *Appl. Phys. Lett.* **110**, 041606 (2017).

[10] J. Zhang *et al.*, "High-Pressure Floating–Zone Growth of Perovskite Nickelate $LaNiO_3$ Single Crystals", *Cryst. Growth Des.* **17**, 2730 (2017).

[11]Guo, H., Z. W. Li, L. Zhao, Z. Hu, C. F. Chang, C-Y. Kuo, W. Schmidt *et al.*, "Antiferromagnetic correlations in the metallic strongly correlated transition metal oxide $LaNiO_3$", *Nat. Commun.* **9**, 43 (2018).

[12] T. Moriya and T. Takimoto, "Anomalous Properties around Magnetic Instability in Heavy Electron Systems", *J. Phys. Soc. Jpn.* **3**, 960 (1995).

[13]R. Hlubina and T. M. Rice, "Resistivity as a function of temperature for models with hot spots on the Fermi surface", *Phys. Rev. B* **52**, 13043 (1995).

[14] A. Rosch, "Interplay of Disorder and Spin Fluctuations in the Resistivity near a Quantum Critical Point", *Phys. Rev. Lett.* **82**, 4280 (1999).

[15] A. Rosch, "Magnetotransport in nearly antiferromagnetic metals", *Phys. Rev. B* **62**, 4945 (2000).

[16] H.-K. Yoo *et al.*, "Latent instabilities in metallic $LaNiO_3$ films by strain control of Fermi-surface topology", *Sci. Rep.* **5,** 8746 (2015).

[17] H. park, A. J. Millis, and C. A. Marianetti. "Site-selective Mott transition in rare-earth-element nickelates." *Phys. Rev. Lett.* **109,** 156402 (2012).

[18] S. Johnston, A. Mukherjee, I. Elfimov, M. Berciu, and G. A. Sawatzky, "Charge disproportionation without charge transfer in the rare-earth-element nickelates as a possible mechanism for the metal-insulator transition." *Phys. Rev. Lett.* **112**, 106404 (2014).

[19] B. Li, D. Louca, S. Yano, L. G. Marshall, J. Zhou, and J. B. Goodenough. "Insulating pockets in metallic $LaNiO_3$." *Adv. Electron. Mater.* **2**, 1500261 (2016).

[20] F. Rivadulla, J.-S. Zhou and J.B. Goodenough, "Electron scattering near an itinerant to localized electronic transition", *Phys. Rev. B* **67**, 165110 (2003).

[21] V. P.-Tinbergen, Tineke, and A. J. Dekker, "Spin-dependent scattering and resistivity of magnetic metals and alloys." *Physica* **29**, 917-937 (1963).

[22] A. C. Hewson, *The Kondo Problem to Heavy Fermions* (Cambridge University Press, 1993).
[23] T. Moriya and K. Ueda, *Rep. Prog. Phys.* **66**, 1299 (2003).
